# Percutaneous Epidural Hydrogel Sealant for the Treatment of Spontaneous Intracranial Hypotension: A Case Report of Chronic Thoracic Neuralgia and Technical Lessons Learned

**DOI:** 10.1155/2018/4189518

**Published:** 2018-07-03

**Authors:** Michael G. Hillegass, Samuel F. Luebbert, Maureen F. McClenahan

**Affiliations:** ^1^Anesthesia & Perioperative Medicine, Medical University of South Carolina, 167 Ashley Avenue, Suite 301, MSC 912, Charleston, SC 29425-9120, USA; ^2^Department of Anesthesiology and Pain Medicine, Naval Medical Center Portsmouth, 620 John Paul Jones Circle, Portsmouth, VA 23708-2197, USA

## Abstract

We report a case in which a 34-year-old female with refractory intracranial hypotension headaches due to a spontaneous dural tear was ultimately treated with CT-guided transforaminal epidural placement of a synthetic absorbable sealant (DuraSeal®). The procedure successfully resolved her headaches; however she subsequently developed thoracic neuralgia presumably due to mass effect of the sealant material on the lower thoracic spinal cord and nerve roots. This case report describes the potential for significant spinal cord and nerve root compression as well as the development of chronic neuralgia with the placement of epidural hydrogel and fibrin glue sealants. Careful consideration should be taken into the needle gauge, needle position, injectate volumes, and injection velocity when delivering the sealant to the epidural space. Use of an 18-gauge Tuohy needle with a slow but steady injection pressure, constant patient feedback, and a conservative injectate volume (less than 2 ml per level) may best optimize sealant delivery to minimize the risk of spinal cord compression and neurologic injury.

## 1. Introduction

Spontaneous intracranial hypotension, once considered rare, can result from spontaneous cerebrospinal fluid (CSF) leak. It is now recognized as an important and more common cause of chronic daily headaches [1]. Percutaneous epidural blood patch placement, often multiple, at either distant or targeted sites has become a recognized treatment for those that have failed conservative measures. However, the efficacy of this technique is variable and has approximated to be only 30% [2]. Neurosurgical repair is reserved for severely debilitating and refractory cases [3]. Epidural placement of fibrin sealant, which forms a fibrin polymer complex through the mixture of fibrinogen and thrombin with a calcium cofactor, has been used successfully in the treatment of intractable postural headaches due to spontaneous CSF leaks [4]. DuraSeal is a polyethylene glycol-based hydrogel sealant that is also used for repair of dural tears. In review of the literature regarding this technique, few complications have been reported and technical guidance with respect to percutaneous placement of dural sealants is inconsistent and limited (Table 1).

The objective of this case report is to highlight technical lessons learned and report a postprocedural adverse event of chronic thoracic neuralgia. We intend to expound on the critical lessons learned regarding needle size, needle tip placement, rate of injection of sealant, and meaningful endpoints to injectate volume that will be useful to clinicians considering this treatment modality. It is our hope that this report will ultimately contribute to the safe and effective delivery of this valuable surgical sparing technique for other patients.

## 2. Case Description

**Figure 1:**
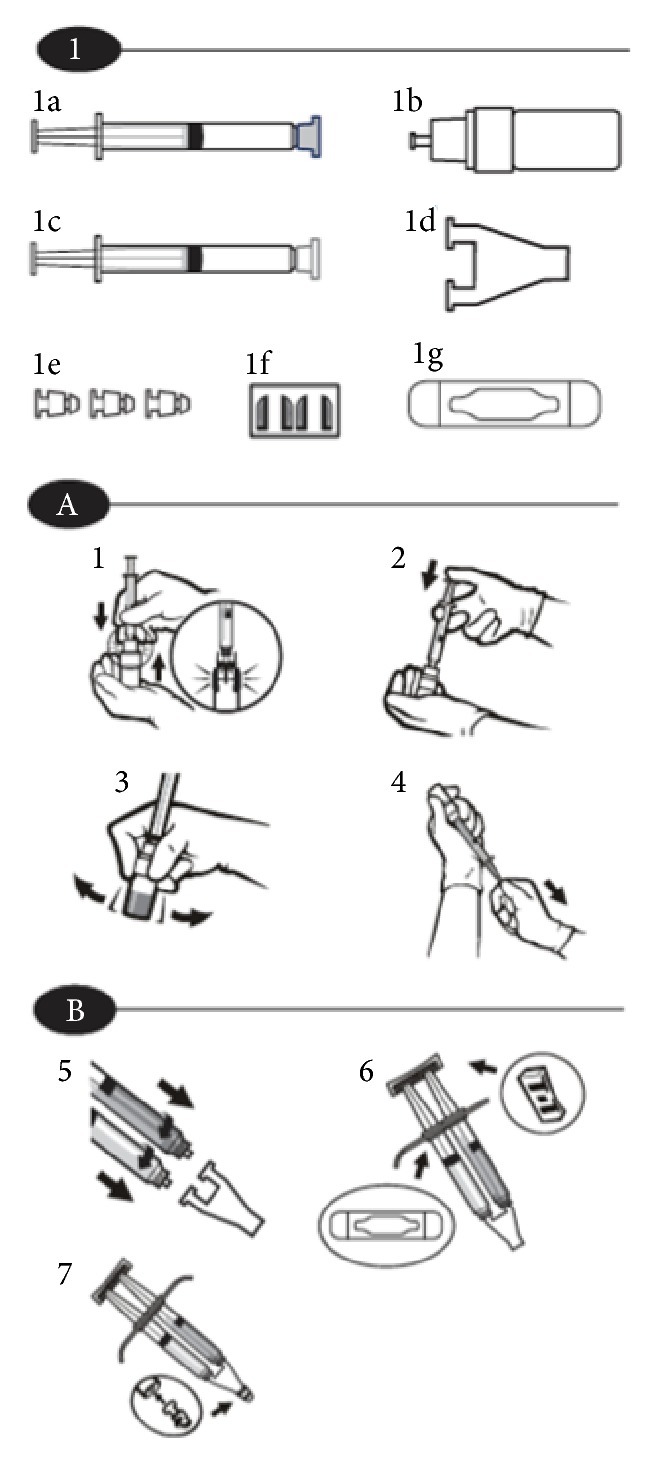
Schematic of DuraSeal dural sealant system assembly.* Note*. Omit step 7 as the spray tip is not needed for percutaneous injection of the hydrogel sealant. Instead attach the applicator directly to the Tuohy needle hub using the Luer lock mechanism.

**Figure 2:**
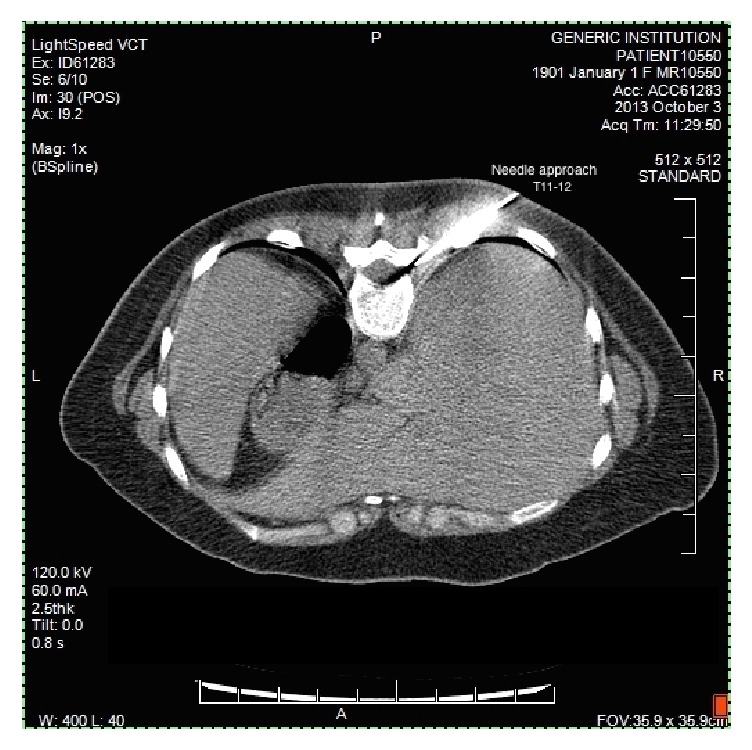
CT-guided image displaying the Tuohy needle trajectory to achieve right-sided transforaminal epidural placement for the injection of hydrogel sealant.

We present a 34-year-old female with a radiographically confirmed anterior dural tear at T10–T12 on MRI causing refractory spontaneous intracranial hypotension headaches. She had failed multiple epidural blood patch placements (6 total) over a three-month period. Her headaches were severely disabling, adversely affecting her quality of life and prevented her from working. The headaches required her to remain mostly recumbent for symptom palliation. A CT-guided percutaneous epidural placement of a synthetic absorbable sealant (DuraSeal, Confluent Surgical, Inc., Waltham, MA) using a right transforaminal approach at T10-T11 and T11-T12 was planned. The hydrogel sealant was prepared according to package insert instructions (Figure 1). After placement of a 20-gauge Tuohy needle at the T11-T12 level, 2.5 ml of sealant was slowly injected. The goal volume of 4 ml was not achieved secondary to plugging of the material within the needle. Next an 18-gauge Tuohy needle was used for transforaminal placement of the sealant at T10-T11 with the goal volume of 5 ml. The final needle tip location was placed slightly more ventral (Figure 2) compared to the initial injection at T11-12 as the dural defect had previously been identified to be more ventral in location. In addition to the larger gauge Tuohy needle, a faster injection rate was used at this level in order to avoid premature plugging of the hydrogel material within the needle. The patient experienced severe localized back pain towards the end of the target injectate volume.

**Figure 3:**
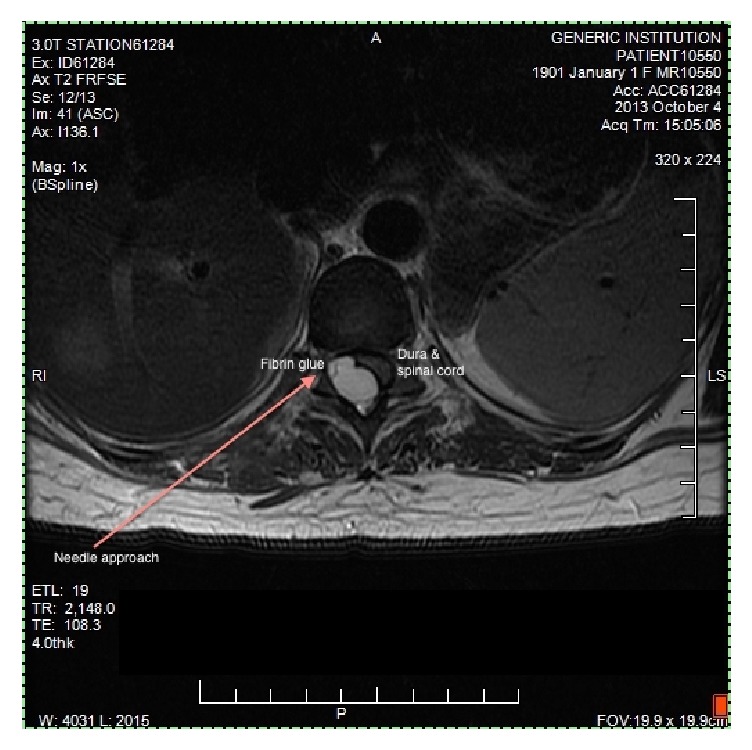
Axial T2 postinjection MRI demonstrating extradural hydrogel sealant deposition with leftward displacement of the thecal sac and spinal cord at T10-11.

Immediate postprocedure CT images were obtained. At the T11-T12 level, a substantial portion of the hydrogel complex was located along the exiting spinal nerve root. At T10-T11, the majority of the volume resided extradural within the spinal canal resulting in significant leftward displacement of the spinal cord. The patient remained without signs of neurologic compromise throughout an extended observation period. She was discharged home with postprocedure instructions and oxycodone for pain control. The patient presented to the Emergency Department with unremitting back pain later the same day and was admitted for pain control and observation. A MRI of the spine showed hydrogel sealant material impression on the right dorsolateral surface of the spinal cord at the T10-T11 level with normal cord signal (Figure 3). The patient subsequently developed signs and symptoms consistent with a concordant right-sided dermatomal thoracic neuralgia, presumably due to mass effect on the lower thoracic spinal nerve roots, but did not demonstrate any myelopathy. She was started on gabapentin and amitriptyline and continued on oxycodone for breakthrough pain. The patient's postural headache completely resolved within hours of the procedure. She was discharged home after a three-day hospital stay. She was able to return to work after four months total of short-term disability, which accounted for her entire evaluation and treatment period. Five months after the procedure the patient remained headache free, but she continued to suffer from chronic mild thoracic neuralgia. Her neuropathic symptoms steadily improved and were well controlled with topiramate, amitriptyline, and as needed tramadol.

## 3. Discussion

This case report describes the potential for significant spinal cord compression as well as the development of chronic thoracic neuralgia with the placement of epidural sealants. Notably, DuraSeal hydrogel sealant can expand by 50% after delivery and last 4 to 8 weeks* in vivo* [14], which may have serious implications within the confined spinal canal when considering the possibility of spinal cord or nerve root compression. Injectate volumes of 3 to 5 ml have commonly been reported in the literature for various fibrin sealants (Table 1), although in some cases as much as 20 ml total (4-5 ml per level) has been used in the transforaminal approach [11]. There were no case reports found with DuraSeal volumes. Given the propensity for expansion of DuraSeal, we recommend that a goal volume of 2 ml or less of injectate should be used per level with the transforaminal approach in order to minimize spinal cord and nerve root compression. Consideration of using smaller volumes (approximately 1 ml) with bilateral injections at the same level and more ventrally placed needle tips should be made to further decrease this risk. Patient self-report of back or radicular pain should be used to terminate the injection before the goal volume is achieved, especially if there is risk of volume expansion of the injectate as is the case with DuraSeal hydrogel. The time to peak volume expansion has been found between days 3 and 14 for DuraSeal [15] so the patient should be educated on the potential of this adverse effect.

The use of 16-, 18-, and 20-gauge needles for sealant injection has been described in the literature (Table 1). In this case, the use of a 20-gauge Tuohy needle and slow rate of injection resulted in premature coagulation of sealant within the needle bore. The use of an 18-gauge Tuohy needle and increased rate of injection prevented the premature coagulation of sealant. However, the increase in injection velocity likely contributed to the significant displacement of the spinal cord by the hydrogel mass. The needle tip at T10-T11 was located several millimeters more ventral and medial within the spinal canal compared to the placement at T11-T12. This needle placement difference also likely contributed to the different distributions of the hydrogel mass.

In summary, the use of an 18-gauge needle should allow for a slow rate of injection without premature coagulation of sealant within the needle bore. This will enable the physician to administer the target volume in a controlled fashion and also use clinically meaningful endpoints for injectate volume, which should aid in minimizing the risk of neurologic injury. These technical points are important to note given the potential for postinjection sealant volume expansion (in the case of DuraSeal) and the resultant risk for spinal cord compression and ischemia. It is also important to note that the different synthetic sealant and fibrin glue products used as dural sealants have varying properties and design assemblies that may make them more or less amenable for percutaneous use. These details should be thoroughly reviewed prior to their application in similar cases.

## Figures and Tables

**Table 1:** Summary of literature review for epidural placement of fibrin glue. CSF, cerebrospinal fluid; IDDS, intrathecal drug delivery system; SIH, spontaneous intracranial hypotension; PDPH, post dural puncture headache; CT, computed tomography; Fluoro, fluoroscopy; TF, transforaminal; IL, interlaminar; LOR, loss of resistance; NR, not reported.

Source	Diagnosis	Number of patients	Sealant	Volume (ml)	Needle gauge	Level	Approach	Guidance	Complications
Gerritse et al. [5]	CSF leak after IDDS implant	3	Tissucol, duo 500, Immuno-AG	4 4 3	18	Lumbar	NR	NR	NR

Trentman et al. [6]	SIH	1	Tisseel, Baxter	3.5 5	18	T4-5 T11-12	TF	CT	NR

Gladstone et al. [7]	SIH	1	Tisseel, Baxter	3.5 4	18	T2 T3	TF	CT	NR

Kamada et al. [8]	SIH	1	Beriplast P, Aventis Pharma	2.8	16	C2	Catheter	N/A	NR

Patel et al. [9]	Post-surgical leak	23	Autologous CRYO-thrombin-CaCl mixture	4–24	18–20	Lumbar, thoracic, cervical, skull base	NR	CT	Aseptic meningitis, *n* = 5

Crul et al. [10]	PDPH	1	Tissucol, duo 500, Immuno AG	3	18	L3-4	IL	LOR	NR

Schievink et al. [11]	SIH	4	Tisseel, Baxter	4	18–20	Left T12-L1	TF	CT-cervical, Fluoro-thoracic and lumbar	NR
				16 (4/level)		Bilateral C5-6 Bilateral C6-7			
				20 (4/level)		Bilateral C5-6 Bilateral C6-7 Left C4-5			
				10 (5/level)		Bilateral C6-7			

Wong and Monroe [12]	PDPH	1	Evicel, Ethicon/ J&J	5	18	L1-2	IL	Fluoro	NR

Freeman et al. [13]	CSF leak after IDDS implant	3	NR	3 10 7	18	L2-3 L2-3 L1-2	IL	Fluoro	NR
